# Morphological and Transcriptomic Responses of Three Intestinal Segments Following the Replacement of 50% of Dietary Soybean Meal with Walnut Cake in Finishing Large Diqing Tibetan Pigs

**DOI:** 10.3390/biology15181636

**Published:** 2026-09-16

**Authors:** Kang Zhang, Lixing Wang, Xinpeng Li, Shihao Li, Chuhuan Zhou, Yin Xu, Dawei Yan, Xinxing Dong

**Affiliations:** 1 College of Animal Science and Technology, Yunnan Agricultural University, Kunming 650201, China; 13732717494@163.com (K.Z.); lixinpeng9812@163.com (X.L.); lishihao0628@163.com (S.L.); sithuan@163.com (C.Z.); 15261820820@163.com (Y.X.); 2 Yunnan Province Animal Husbandry Station, Kunming 650224, China; 15911716157@163.com

**Keywords:** intestinal morphology, transcriptome, Diqing Tibetan pig, walnut cake, soybean meal

## Abstract

Alternative feed ingredients may alter intestinal structure and function. We examined jejunal, ileal, and cecal responses in Large Diqing Tibetan pigs fed a control diet or one in which walnut cake replaced 50% of soybean meal. Two pigs were sampled from each of three pens per treatment. Intestinal morphology was assessed histologically, and RNA sequencing data from 8 jejunal, 12 ileal, and 10 cecal samples retained after screening were analyzed by voom–dream, differentially expressed gene (DEG) enrichment analysis, and gene set enrichment analysis (GSEA). The 50W group showed higher villus-related indices in the jejunum and ileum and greater cecal mucosal thickness and crypt depth. The screened-sample analysis identified 103, 67, and 1979 DEGs, respectively. Pathway analyses associated jejunal responses with nutrient metabolism, ion transport, and epithelial organization; ileal responses with DNA replication, cell cycle, and epithelial renewal; and cecal responses with extracellular matrix organization and cell adhesion. Although jejunal and cecal DEG counts were sensitive to sample inclusion, principal pathway-enrichment directions were broadly maintained in full-sample and leave-one-pen-out analyses. RT-qPCR results for five selected genes showed concordant directions of expression change with the corresponding RNA-seq results.

## Simple Summary

Soybean meal is a common protein source in pig diets, but local alternatives could reduce reliance on conventional feeds. Walnut cake, the material left after oil is extracted from walnuts, may be one such alternative. We studied Large Diqing Tibetan pigs fed either a control diet or a diet in which walnut cake replaced half of the soybean meal. We examined three intestinal regions to assess their structure and gene activity, which reflects how cells use their genetic instructions. Compared with pigs fed the control diet, those fed the replacement diet had taller and wider finger-like projections on the inner surface of the two parts of the small intestine examined. These projections help absorb nutrients from food. In a pouch at the beginning of the large intestine, the inner lining was thicker and the small glands within it were deeper. The three intestinal regions showed different patterns of gene activity in response to the replacement diet. These findings provide basic information for further evaluation of walnut cake as a partial replacement for soybean meal in pig diets.

## 1. Introduction

Rising feed costs and pressure on conventional protein supplies have increased interest in alternatives to soybean meal in pig diets [1]. Such ingredients should be evaluated not only for their protein and energy contributions but also for their effects on gastrointestinal structure and function [2]. Because the intestine is central to nutrient digestion and absorption, epithelial barrier maintenance, and host homeostasis [3], changes in its structure and molecular function are relevant to the evaluation of feed suitability. Dietary protein sources can affect intestinal morphology and function in pigs [4,5]. Moreover, the amount of undigested protein and the fermentability of dietary fiber shape the substrates available to the hindgut microbiota and the resulting metabolite profile. Proteolytic fermentation may generate potentially harmful metabolites, whereas fermentation of fermentable dietary fiber produces short-chain fatty acids that support epithelial energy metabolism and barrier integrity [6]. Unfavorable microbial and metabolic changes can impair the epithelial barrier and increase intestinal permeability, resulting in a condition commonly referred to as “leaky gut” [7]. Therefore, these intestinal responses should be considered when assessing alternative protein ingredients.

Walnut cake, a major by-product of walnut oil extraction, contains protein, residual lipids, dietary fiber, polyphenols, and other components, making it a potential alternative feed ingredient [8]. Related walnut-kernel oil-extraction by-products are described in the cited literature as walnut kernel cake, walnut oilcake, or walnut meal, although their processing conditions and composition may differ. For consistency, the term walnut cake is used throughout this manuscript for the ingredient evaluated here. In young pigs, the inclusion of walnut cake at 4–8% was reported to improve feed utilization and economic efficiency without adversely affecting health or blood variables [9]. In adult goats, inclusion of walnut cake at 10% of the concentrate did not affect feed intake, nutrient digestibility, or nitrogen, calcium, and phosphorus balance [10]. In broiler chickens, replacing soybean meal with walnut cake at levels up to 20% did not compromise production performance or nutrient digestibility and improved ileal villus morphology, whereas a 30% replacement impaired performance [11]. These findings suggest that responses to walnut cake may depend on the animal species and dietary inclusion level. Transcriptomic evidence further indicates that walnut cake can alter adipose gene expression in pigs [12]. Our previous study showed that replacing 50% of dietary soybean meal with walnut cake did not affect final body weight or average daily gain but reduced backfat thickness and carcass fat percentage and increased intramuscular fat content in finishing Large Diqing Tibetan pigs [13]. However, that study focused on production traits and fat deposition and did not examine intestinal morphology or transcriptomic responses across individual intestinal segments.

Different segments of the porcine intestine have distinct structural and physiological functions. The jejunum and ileum participate in nutrient digestion and absorption [14], whereas the cecum has distinct hindgut functions and is an important site of microbial fermentation [15]. Differences in nutrient exposure and local physiological environments provide a rationale for analyzing the jejunum, ileum, and cecum separately.

The Diqing Tibetan pig is an indigenous plateau breed adapted to high-altitude environments and characterized by roughage tolerance and a strong capacity for dietary fiber utilization, making the evaluation of locally available fibrous feed resources particularly relevant [16]. Given the different physiological roles of the jejunum, ileum, and cecum, we hypothesized that the predominant morphological and transcriptomic response patterns would vary among these segments. Therefore, this study aimed to characterize the morphological and transcriptomic responses of the jejunum, ileum, and cecum in Large Diqing Tibetan pigs fed either a basal diet or a diet in which walnut cake replaced 50% of soybean meal.

## 2. Materials and Methods

### 2.1. Ethics Statement

The animal experiment was approved by the Animal Ethics Committee of Yunnan Agricultural University (approval no. APYNAU202401001; approval date: 4 January 2024).

### 2.2. Experimental Design and Animal Management

After the results of the previously reported four-treatment, 12-week feeding trial [13] had been evaluated, the 0W and 50W groups were selected for the follow-up histomorphological and transcriptomic analyses reported here. The feeding trial was conducted at the Tibetan pig breeding base of Weixi Wanjia Animal Husbandry Co., Ltd. (Weixi Lisu Autonomous County, Diqing Tibetan Autonomous Prefecture, Yunnan Province, China). The trial included 72 healthy castrated male Large Diqing Tibetan pigs of similar genetic background, aged approximately 6 months and weighing approximately 70 kg at the start of the trial. The use of animals with the same sex and reproductive status was intended to reduce biological variation unrelated to dietary treatment. The pigs were randomly assigned to four dietary treatments, with 18 pigs per treatment housed in three physically separate pens of six pigs each. Pens assigned to the same treatment were spatially grouped, and pen location within the facility was not randomized. The 0W group received the basal diet, whereas the 25W, 50W, and 100W groups received diets in which walnut cake replaced 25%, 50%, and 100% of the soybean meal in the basal diet, respectively. Initial and final body weights for the treatment groups were reported in the original feeding-trial report (Table 3 in [13]). All diets were formulated to be isonitrogenous and isoenergetic in accordance with the Nutrient Requirements of Swine (GB/T 39235–2020) [17]. During diet formulation, the inclusion levels of corn, soybean oil, and supplemental L-lysine were adjusted accordingly to maintain broadly comparable dietary energy, crude protein, and total lysine levels among treatments. Supplemental L-lysine was used to balance dietary nutrient levels and was not treated as an independent experimental factor (Table 1). All pigs were housed under identical conditions. Feed was replenished in feed troughs three times daily at approximately 09:00, 14:00, and 20:00, with a small amount of residual feed maintained to ensure ad libitum access. Water was continuously available through bowl drinkers. Feed intake, residual feed, and herd health were recorded daily. Before the feeding trial, the pigs had been vaccinated against classical swine fever at 20, 60, and 80 days of age and against *Mycoplasma hyopneumoniae* at 7 and 40 days of age, in accordance with the farm’s routine vaccination program. The dietary formulations are presented in Table 1. The walnut cake used in the experimental diets was produced by mechanical pressing at a local processing facility in Weixi Lisu Autonomous County, Diqing Tibetan Autonomous Prefecture, Yunnan Province, China, and stored at ambient temperature in a warehouse before diet preparation. Its analyzed nutrient composition is provided in Table S1.

The ingredient composition and selected nutrient levels were previously reported [13]; additional nutrient values are provided here.

### 2.3. Slaughter and Sample Collection

At the end of the feeding trial, a representative sampling strategy based on final body weight was applied within each of the four dietary groups. Two pigs whose final body weights were closest to the mean final body weight of their respective pen were selected from each pen for slaughter and intestinal sampling. For the follow-up analyses reported here, samples from the 0W and 50W groups were included, resulting in six pigs per group and 12 pigs in total. The 12 pigs selected from the previously reported feeding trial [13] had final body weights of 129.26 ± 2.89 kg in the 0W group and 132.55 ± 3.49 kg in the 50W group (mean ± SD; six pigs per treatment), with no statistically significant difference detected between treatments (*p* = 0.2384). After a 12-h fast with free access to water, the pigs were electrically stunned and slaughtered by cardiac exsanguination. Tissue samples were collected from the middle regions of the jejunum, ileum, and cecum. Samples for RNA sequencing and reverse transcription quantitative PCR (RT-qPCR) were rinsed with physiological saline, snap-frozen in liquid nitrogen, and stored at −80 °C; samples for morphological analysis were fixed in 4% paraformaldehyde. All samples were collected from the same anatomical locations using identical procedures.

### 2.4. Measurement of Intestinal Morphology

Fixed jejunal, ileal, and cecal tissues were dehydrated through a graded ethanol series (Guangzhou Chemical Reagent Factory, Guangzhou, Guangdong, China), cleared in xylene (Chongqing Chuandong Chemical (Group) Co., Ltd., Chongqing, China), embedded in paraffin (Leica Biosystems, Nussloch, Baden-Württemberg, Germany), and sectioned at 6 μm using a KD-2260 rotary microtome (Zhejiang Jinhua Kedi Instrumental Equipment Co., Ltd., Jinhua, Zhejiang, China). Paraffin sections were dried at 60 °C for 1 h, deparaffinized in xylene, and rehydrated through a descending ethanol series. The sections were stained with hematoxylin for 5 min, rinsed with water, differentiated in hematoxylin differentiation solution for 1–3 s, rinsed under running tap water, and counterstained with eosin for 10 s. Subsequently, the sections were dehydrated through an ascending ethanol series, cleared in xylene, and mounted with neutral balsam. Hematoxylin, eosin, and hematoxylin differentiation solution were obtained from Wuhan Servicebio Technology Co., Ltd. (Wuhan, Hubei, China), and neutral balsam was obtained from Sinopharm Chemical Reagent Co., Ltd. (Shanghai, China). The stained sections were examined and imaged using an RX50 light microscope (Ningbo Sunny Instruments Co., Ltd., Yuyao, Zhejiang, China), and morphological parameters were measured using Image-Pro Plus 6.0 (Media Cybernetics, Rockville, MD, USA). The observer performing the morphometric measurements was not blinded to dietary treatment. For each pig, three histological sections from each intestinal segment were evaluated. For the jejunum and ileum, all intact, well-oriented villus–crypt units with clearly defined boundaries across the three sections were measured, yielding at least 10 valid units per pig. Villus height (VH), crypt depth (CD), and villus width (VW) were measured, and the villus height-to-crypt depth ratio (V/C) was calculated. For the cecum, all intact regions with clearly defined boundaries across the three sections were measured, yielding at least 10 valid regions per pig. Mucosal thickness (MT), muscularis mucosae thickness (MM), muscularis propria thickness (MP), and CD were measured. All thickness measurements were made perpendicular to the corresponding tissue layers. Cecal mucosal thickness was measured from the luminal surface to the upper boundary of the muscularis mucosae; muscularis mucosae thickness was measured between its upper and lower boundaries; and muscularis propria thickness was measured between its inner and outer boundaries. For each pig, the mean of all valid measurements was calculated for each parameter.

### 2.5. RNA Extraction, Library Construction, and Sequencing

Total RNA was extracted from jejunal, ileal, and cecal tissues using TRIzol reagent (Invitrogen, Carlsbad, CA, USA). RNA concentration and purity were assessed using a NanoDrop ND-1000 spectrophotometer (NanoDrop Technologies, Wilmington, DE, USA), and RNA integrity was evaluated using an Agilent 2100 Bioanalyzer (Agilent Technologies, Santa Clara, CA, USA) and agarose gel electrophoresis. Samples with an RNA concentration > 50 ng/μL, an RNA integrity number > 7.0, an OD260/280 ratio > 1.8, and a total RNA amount > 1 μg were used for library construction. For each intestinal segment, samples from six pigs per treatment were initially subjected to library construction and sequencing.

Poly(A) + mRNA was isolated by two rounds of purification using Dynabeads Oligo(dT) (Thermo Fisher Scientific, Waltham, MA, USA) and fragmented at 94 °C for 5–7 min using the NEBNext Magnesium RNA Fragmentation Module (New England Biolabs, Ipswich, MA, USA). Strand-specific libraries were constructed using the dUTP method, followed by end repair, A-tailing, adapter ligation, size selection, uracil-DNA glycosylase digestion, and PCR amplification. Libraries with an insert size of 300 ± 50 bp were subjected to 150-bp paired-end sequencing on an Illumina NovaSeq 6000 platform (Illumina, San Diego, CA, USA) by LC-Bio Technology Co., Ltd. (Hangzhou, China). All samples were submitted together for RNA extraction, library preparation, and sequencing. Statistical models did not include a batch covariate because laboratory batch information was unavailable.

### 2.6. Sequencing Data Quality Control, Alignment, and Gene Quantification

Raw reads were processed using fastp (version 1.0.1) [18] to remove adapter-containing reads, reads with more than five N bases, low-quality reads, and reads shorter than 30 bp. Clean reads were aligned to the Sscrofa11.1 reference genome using HISAT2 (version 2.2.1) [19], and mapping rates were calculated. Gene-level counts for differential expression analysis were generated using featureCounts (version 2.1.1) in paired-end, reverse-stranded mode, with exons as features and gene_id as the grouping attribute, based on the Ensembl Sscrofa11.1 release 110 annotation [20]. Quality-control and alignment statistics for all RNA-seq libraries, including clean-read counts, Q20 and Q30 values, GC content, overall mapping rates, unique mapping rates, and fastp-estimated duplication rates, are provided in Table S2.

### 2.7. Sample Screening, Principal Component Analysis, and Differential Expression Analysis

Before downstream transcriptomic analyses, unsupervised hierarchical clustering was performed separately for each intestinal segment using variance-stabilized expression profiles generated with DESeq2 (version 1.46.0) [21] to assess sample-level expression similarity. Based on the resulting dendrograms, four jejunal samples and two cecal samples showing distinct sample-level clustering patterns were excluded from the primary screened-sample analyses, whereas all ileal samples were retained. After screening, 8 jejunal samples (4 per group), 12 ileal samples (6 per group), and 10 cecal samples (6 from the 0W group and 4 from the 50W group) were retained for subsequent transcriptomic analyses. The retained datasets represented four pens in the jejunum (two per treatment), six in the ileum (three per treatment), and five in the cecum (three in the 0W group and two in the 50W group). Excluded sample identifiers and pen-level retention summaries are provided in Table S3A and S3B, respectively, whereas the original dendrograms and pre-exclusion PCA plots are shown in Figures S1 and S2, respectively.

Transcripts per million (TPM) values were calculated from raw counts and gene lengths. Genes with TPM ≥ 1 in at least one sample in the corresponding PCA dataset were log_2_(TPM + 1)-transformed for principal component analysis. For differential expression analysis, genes with raw counts ≥ 10 in at least two retained samples were retained. After this filtering, 16,299 jejunal, 17,352 ileal, and 16,397 cecal genes were retained in the screened-sample analyses. Raw count matrices were converted to DGEList objects and normalized using the trimmed mean of M-values method in edgeR (version 4.0.16) [22]. The mean–variance relationship was modeled using precision weights estimated with voomWithDreamWeights in variancePartition (version 1.32.5), based on the voom framework in limma (version 3.58.1) [23]. Gene-wise linear mixed-effects models were then fitted using dream [24]. All corresponding gene-wise models converged successfully, and no genes were excluded because of model-fitting failure. Treatment was included as a fixed effect and pen as a random intercept to account for pigs sampled from the same pen. Kenward–Roger small-sample inference was followed by empirical Bayes moderation. *p* values were adjusted using the Benjamini–Hochberg method, and genes with |log_2_FC| ≥ 1 and FDR < 0.05 were considered differentially expressed. The FDR threshold was used to define statistical significance, whereas |log_2_FC| ≥ 1 was applied separately as a minimum fold-change criterion. The same analytical workflow and DEG criteria were applied in the full-sample sensitivity analyses of the jejunum and cecum.

### 2.8. Functional Enrichment Analysis

All differentially expressed genes identified in the screened-sample analysis and meeting the above criteria, including both upregulated and downregulated genes, were analyzed together using Gene Ontology (GO) and Kyoto Encyclopedia of Genes and Genomes (KEGG) over-representation analyses in KOBAS-i (http://bioinfo.org/kobas; accessed on 3 September 2025) [25], with *Sus scrofa* selected as the reference species. The default *Sus scrofa* background provided by KOBAS-i was used for the over-representation analyses. Enrichment was evaluated using the default hypergeometric test/Fisher’s exact test, and *p* values were adjusted using the Benjamini–Hochberg method. Terms or pathways with a corrected *p* value < 0.05 were considered significantly enriched. Complete GO and KEGG over-representation results are provided in Tables S4 and S5, respectively. For visualization in the main text, only significant GO biological process terms were retained.

### 2.9. Gene Set Enrichment Analysis

Gene set enrichment analysis (GSEA) was performed separately for the jejunum, ileum, and cecum using all genes retained after the count-based filtering applied in the corresponding differential expression analyses [26]. Ensembl gene identifiers were mapped to Entrez Gene identifiers using the biomaRt package (version 2.58.2) and Ensembl release 110 [27]. Exact duplicate mappings were removed, and Ensembl identifiers with one-to-many mappings to Entrez Gene identifiers were excluded. When multiple Ensembl identifiers mapped to the same Entrez Gene identifier, the record with the largest absolute moderated t statistic was retained. Among the count-filtered Ensembl identifiers, 13,958 of 16,299 (85.64%), 14,292 of 17,352 (82.37%), and 13,773 of 16,397 (84.00%) were retained as unique Entrez Gene identifiers for the jejunum, ileum, and cecum, respectively; the corresponding losses during mapping and ambiguity resolution were 2341 (14.36%), 3060 (17.63%), and 2624 (16.00%), respectively. The resulting unique Entrez Gene identifiers were ranked in descending order according to the moderated t statistic from the dream model for the 50W versus 0W contrast. GSEA was performed for all Gene Ontology categories and Kyoto Encyclopedia of Genes and Genomes pathways using the gseGO and gseKEGG functions, respectively, in the clusterProfiler package (version 4.10.1) [28]. GO annotations were obtained from the org.Ss.eg.db package (version 3.22.0). Enrichment testing was performed using the fgsea algorithm implemented in the fgsea package (version 1.28.0). The KEGG organism code was set to “ssc”, the Benjamini–Hochberg method was used for multiple-testing correction, and eps was set to 0. Gene sets containing 10–500 genes were tested with a weighting exponent of 1. The internal *p*-value cutoff was set to 1 to retain all tested gene sets for subsequent filtering. Gene sets with |normalized enrichment score (NES)| > 1, nominal *p* value < 0.05, and FDR < 0.05 were considered significantly enriched. A positive NES indicated enrichment toward the 50W-associated end of the ranked gene list, whereas a negative NES indicated enrichment toward the 0W-associated end.

For the multi-curve enrichment plots in the main text, candidate gene sets were first restricted to those meeting the prespecified significance criteria. Within each intestinal segment and database, significant gene sets were grouped according to shared biological functions, and non-redundant representatives were selected to cover the principal functional themes. Both positive- and negative-NES gene sets were displayed when significant results were available in both directions. Complete ranked screened-sample GO- and KEGG-based GSEA results, including gene sets not displayed in the main figures, are provided in Tables S6 and S7, respectively. Enrichment curves were generated using enrichplot (version 1.22.0). For KEGG pathway visualization, one representative pathway showing significant enrichment toward the 50W-associated end of the ranked gene list was selected for each intestinal segment. Gene-level log_2_ fold changes estimated by the dream model were then mapped onto the selected KEGG pathway diagrams using pathview (version 1.42.0) [29]. When multiple gene entries mapped to the same Entrez Gene identifier, the entry with the largest absolute log_2_ fold change was retained, and the color scale was truncated at ±2. For sensitivity analysis, GSEA was repeated for the jejunum and cecum using the moderated t statistics from the corresponding full-sample dream models, with all other gene-filtering, identifier-mapping, ranking, and enrichment parameters unchanged. Concordance with the screened-sample analyses was evaluated using Pearson correlations of matched NES values and the overlap and directional agreement of significant gene sets. Complete full-sample GO- and KEGG-based GSEA results are provided in Tables S6 and S7, respectively, whereas the full-sample differential-expression results and the corresponding screened-versus-full comparison metrics are provided in Table S8. To evaluate whether the pathway-level results were disproportionately influenced by any single pen, leave-one-pen-out sensitivity analyses were conducted separately for each intestinal segment using the full-sample datasets, with both samples from one pen omitted at a time. The original count-filtering, TMM-normalization, voom–dream modeling, ranking, and GSEA procedures were retained, and stability was assessed from correlations of matched NES values and directional consistency across pen omissions. Complete leave-one-pen-out sensitivity-analysis results are provided in Table S9.

### 2.10. Reverse Transcription Quantitative PCR

Five genes identified as DEGs in the screened-sample analysis—*FGFBP1*, *AMIGO2*, and *ARHGEF25* from the cecum, *ASIC5* from the jejunum, and *CCNB1* from the ileum—were selected for RT-qPCR analysis to assess whether their expression trends were consistent with the RNA-seq results. *GAPDH* was selected as the internal reference gene based on its previous use in RT-qPCR analyses of porcine intestinal tissues [30] and its reported expression stability in minipig intestinal tissue [31]. For each treatment, one RNA-sequenced pig from each pen was selected for RT-qPCR, yielding three pen-associated biological samples per treatment; RT-qPCR was performed using samples from the same pigs included in the RNA-seq analysis. For each sample, 2 μg of total RNA was reverse-transcribed using the FastKing One Step Reverse Transcription Kit (TIANGEN Biotech Co., Ltd., Beijing, China) according to the manufacturer’s instructions. Primers were designed using NCBI Primer-BLAST (https://www.ncbi.nlm.nih.gov/tools/primer-blast/; accessed on 23 October 2025) and synthesized by Kunming Sangon Biotech Co., Ltd. (Kunming, China). The primer sequences are listed in Table 2.

Reactions were performed using SuperReal PreMix Plus (SYBR Green; FP205, TIANGEN Biotech Co., Ltd., Beijing, China) on an FQD-96A real-time PCR detection system (Bioer, Hangzhou, China). Each 20 μL reaction contained 10 μL of 2× SuperReal PreMix Plus, 0.6 μL of each primer (10 μM), 1 μL of cDNA, 0.5 μL of 50× ROX Reference Dye, and 7.3 μL of RNase-free water. Cycling conditions were 95 °C for 15 min, followed by 40 cycles of 95 °C for 10 s and 60 °C for 20 s. Melting curves were generated from 60 °C to 95 °C, and each primer pair used in the final assays yielded a single melting-curve peak. Three technical replicates were included per sample, and relative expression was calculated using the 2^−ΔΔCt^ method [32]. For comparison with the RT-qPCR results, TPM values for these five genes were extracted from the RNA-seq data of the same six pigs used for RT-qPCR, and the mean TPM value was calculated for each treatment.

### 2.11. Statistical Analysis

Statistical analyses of the final body weights of the sampled pigs and intestinal morphology, as well as visualization of the morphological data, were performed using R software (version 4.3.3; R Foundation for Statistical Computing, Vienna, Austria). The final body weights of the 12 sampled pigs were summarized as mean ± SD based on six pigs per treatment. For the between-treatment comparison of final body weight, the final body weights of the two pigs sampled from the same pen were averaged, and each pen mean was used as the experimental unit, resulting in three pen-level observations per treatment. For intestinal morphology, the individual means of the two pigs sampled from the same pen were averaged, and each pen mean was likewise used as the experimental unit for group comparisons. Between-treatment differences were evaluated using Welch’s independent two-sample *t*-test. Effect sizes for morphological outcomes were expressed as mean differences between treatments (50W − 0W), with 95% confidence intervals calculated using the Welch–Satterthwaite method. For graphical presentation, morphological data are shown as mean ± SD of the pen-level observations. The *p* values from all 12 morphological comparisons across the three intestinal segments were adjusted jointly using the Benjamini–Hochberg method to control the false discovery rate (FDR), and differences were considered significant at FDR < 0.05. The 12 prespecified comparisons were treated as a single family because they collectively addressed the overall intestinal morphological response to the dietary treatment across the three segments.

## 3. Results

### 3.1. Changes in Intestinal Morphology

**Figure 1:**
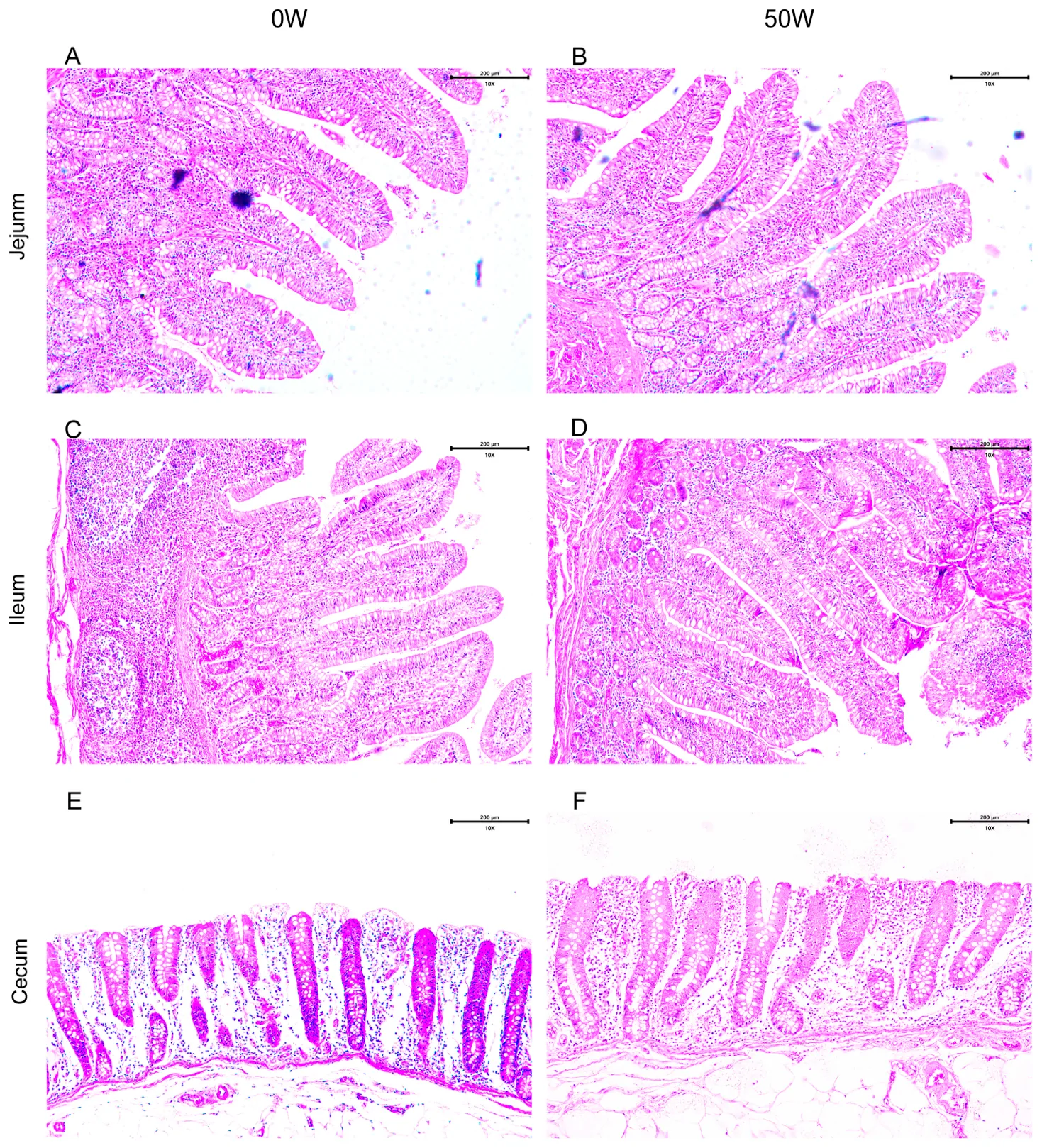
Effects of replacing 50% of dietary soybean meal with walnut cake on the morphology of different intestinal segments in Large Diqing Tibetan pigs. Representative hematoxylin and eosin (H&E)-stained sections of the jejunum (**A**,**B**), ileum (**C**,**D**), and cecum (**E**,**F**). (**A**,**C**,**E**) represent the 0W group, whereas (**B**,**D**,**F**) represent the 50W group. 0W, basal-diet control; 50W, group in which walnut cake replaced 50% of dietary soybean meal. All panels were acquired at the same magnification, and identical 200-μm scale bars are shown in each panel.

**Figure 2:**
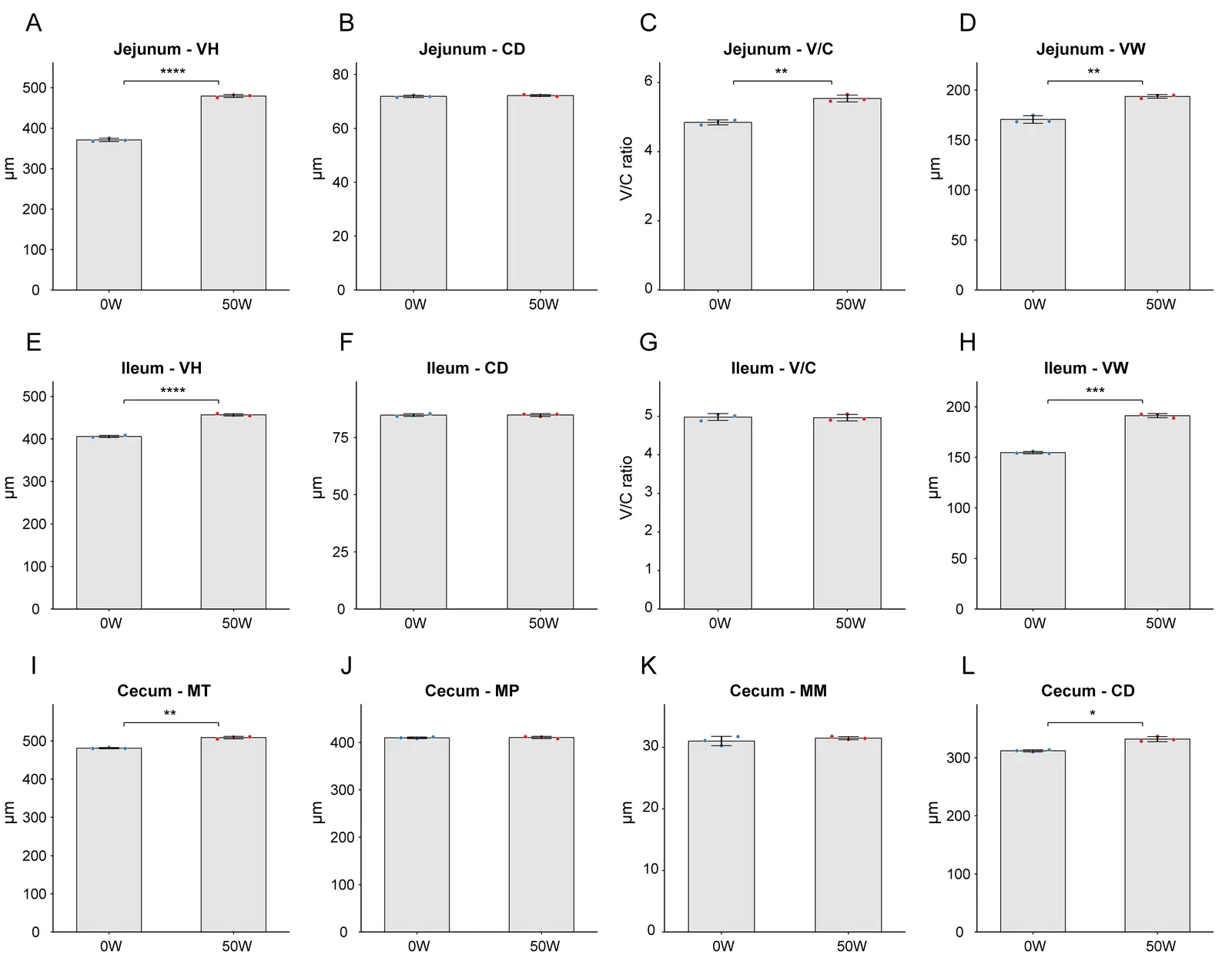
Effects of replacing 50% of dietary soybean meal with walnut cake on morphological indices of the jejunum, ileum, and cecum in Large Diqing Tibetan pigs. (**A**–**D**) Jejunal morphological indices, including villus height (VH), crypt depth (CD), the villus height-to-crypt depth ratio (V/C), and villus width (VW); (**E**–**H**) ileal morphological indices, including VH, CD, V/C, and VW; (**I**–**L**) cecal morphological indices, including mucosal thickness (MT), muscularis propria thickness (MP), muscularis mucosae thickness (MM), and CD. Blue and red points represent pen-level observations for the 0W and 50W groups, respectively, with each point calculated as the mean of the two pigs sampled from the same pen (*n* = 3 pens per treatment). Gray bars and error bars represent the mean and SD of the three pen-level observations, respectively. The *p* values from all 12 morphological comparisons were adjusted jointly using the Benjamini–Hochberg method and are reported as false discovery rate (FDR) values. * indicates FDR < 0.05; ** indicates FDR < 0.01; *** indicates FDR < 0.001; and **** indicates FDR < 0.0001. 0W, basal-diet control; 50W, group in which walnut cake replaced 50% of dietary soybean meal.

Examination of H&E-stained sections, together with quantitative histomorphometric analysis, revealed morphological differences between the 0W and 50W groups (Figure 1 and Figure 2). In the jejunum, villus height (VH; FDR < 0.0001), the villus height-to-crypt depth ratio (V/C; FDR < 0.01), and villus width (VW; FDR < 0.01) were significantly higher in the 50W group, whereas crypt depth (CD) did not differ significantly between groups (FDR ≥ 0.05). In the ileum, VH (FDR < 0.0001) and VW (FDR < 0.001) were significantly higher in the 50W group, whereas CD and V/C did not differ significantly (FDR ≥ 0.05). In the cecum, mucosal thickness (MT; FDR < 0.01) and CD (FDR < 0.05) were significantly higher in the 50W group, whereas muscularis mucosae thickness (MM) and muscularis propria thickness (MP) did not differ significantly (FDR ≥ 0.05). Mean differences between treatments and their 95% confidence intervals, calculated from the pen-level observations, are provided in Table S10.

### 3.2. Changes in Transcriptomic Profiles

**Figure 3:**
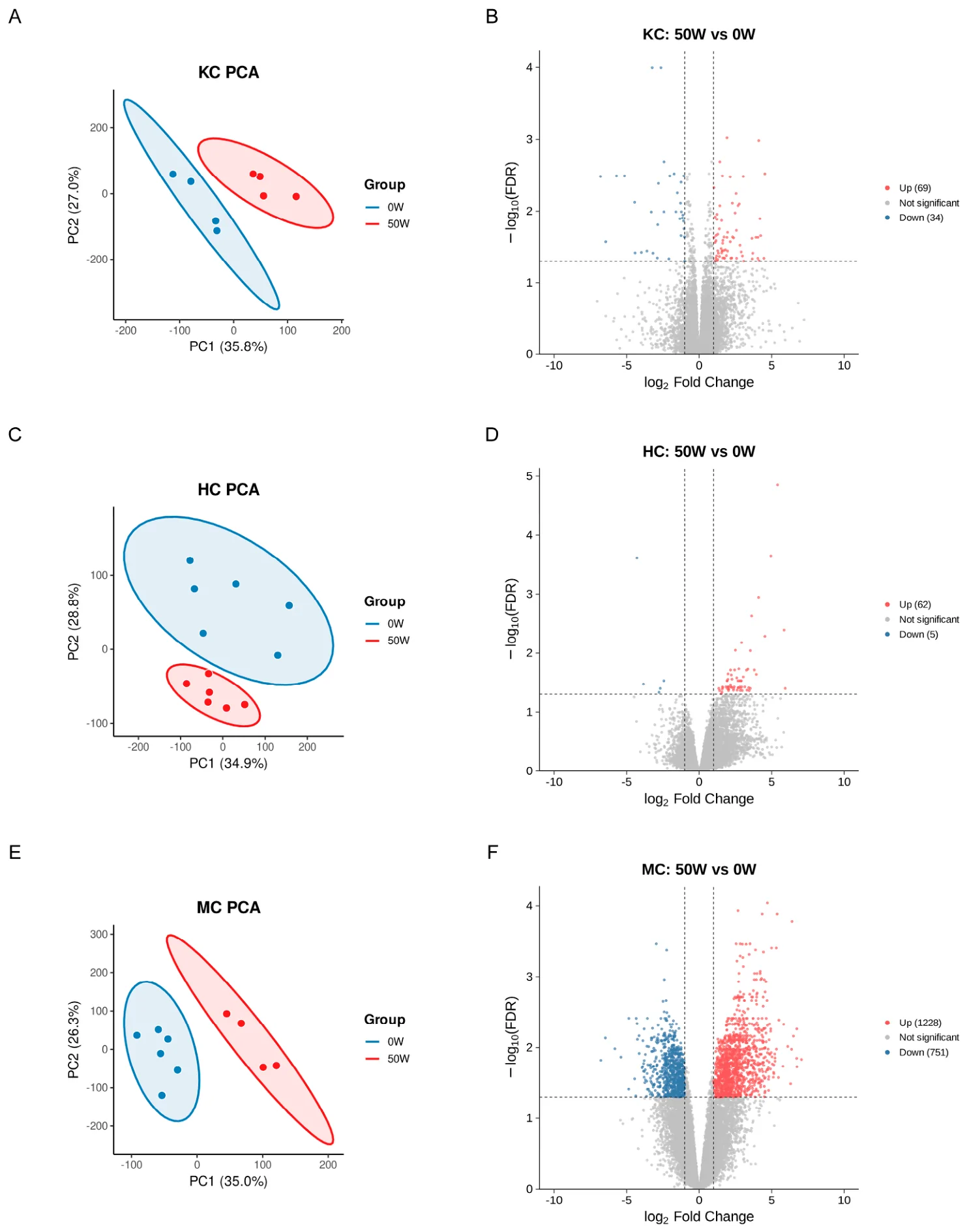
Overall transcriptomic expression patterns and differential expression profiles in different intestinal segments of Large Diqing Tibetan pigs after replacing 50% of dietary soybean meal with walnut cake. (**A**,**C**,**E**) Principal component analysis (PCA) of the RNA-seq samples retained following sample screening based on unsupervised hierarchical clustering in the jejunum, ileum, and cecum, respectively. (**B**,**D**,**F**) Volcano plots of differentially expressed genes between the 50W and 0W groups based on the same retained sample sets in the jejunum, ileum, and cecum, respectively. Eight jejunal samples (0W, *n* = 4; 50W, *n* = 4), 12 ileal samples (0W, *n* = 6; 50W, *n* = 6), and 10 cecal samples (0W, *n* = 6; 50W, *n* = 4) were included. The ellipses in the PCA plots represent 95% data ellipses based on a multivariate t distribution. Red and blue indicate genes upregulated and downregulated, respectively, in the 50W group relative to the 0W group, whereas gray indicates genes without significant differential expression. The criteria for differential expression were FDR < 0.05 and |log_2_FC| ≥ 1. RNA-seq, RNA sequencing; FDR, false discovery rate; log_2_FC, log_2_ fold change; KC, jejunum; HC, ileum; MC, cecum; 0W, basal-diet control; 50W, group in which walnut cake replaced 50% of dietary soybean meal.

Following sample screening, 8 jejunal samples (0W, *n* = 4; 50W, *n* = 4), 12 ileal samples (0W, *n* = 6; 50W, *n* = 6), and 10 cecal samples (0W, *n* = 6; 50W, *n* = 4) were retained for analysis. Principal component analysis of these samples showed an overall tendency toward separation between the 0W and 50W groups in all three intestinal segments (Figure 3A,C,E). Using the criteria |log_2_FC| ≥ 1 and FDR < 0.05, 103 differentially expressed genes (DEGs) were identified in the jejunum, including 69 upregulated and 34 downregulated genes; 67 DEGs were identified in the ileum, including 62 upregulated and 5 downregulated genes; and 1979 DEGs were identified in the cecum, including 1228 upregulated and 751 downregulated genes (Figure 3B,D,F). In the full-sample sensitivity analysis, only two jejunal genes and no cecal genes met the combined criteria of FDR < 0.05 and |log_2_FC| ≥ 1, indicating that the threshold-defined DEG results in these two segments were sensitive to sample inclusion (Table S8).

### 3.3. Functional Enrichment Patterns of Differentially Expressed Genes

**Figure 4:**
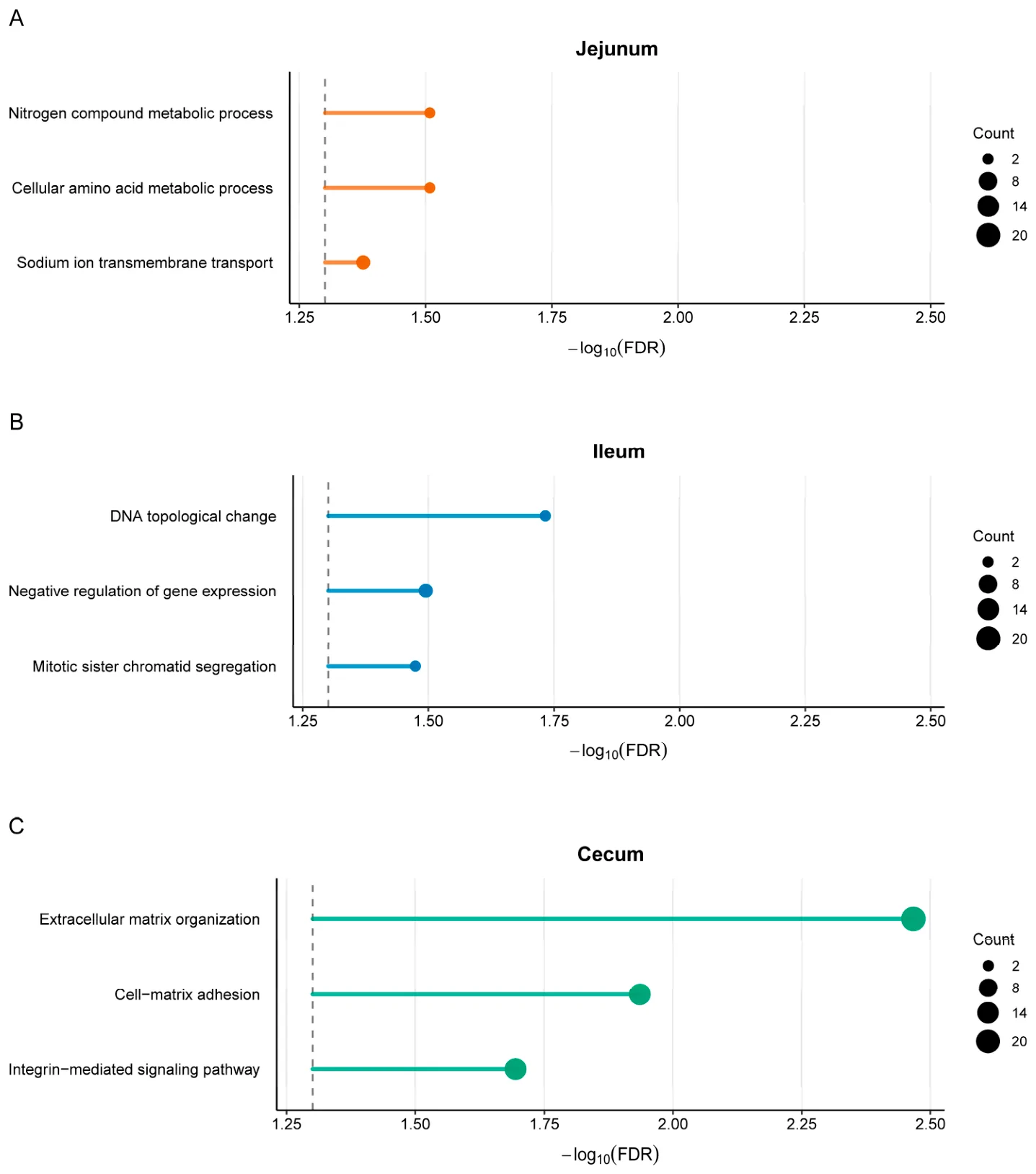
Gene Ontology biological process enrichment analysis of differentially expressed genes between the 50W and 0W groups in the jejunum (**A**), ileum (**B**), and cecum (**C**) of Large Diqing Tibetan pigs. Only terms with a false discovery rate (FDR) < 0.05 are shown. The *x*-axis represents −log_10_(FDR), and circle size indicates the number of differentially expressed genes associated with each term. The “Count” legend provides reference values for the common circle-size scale across the three panels; not every labeled reference value occurs in each panel. 0W, basal-diet control; 50W, group in which walnut cake replaced 50% of dietary soybean meal. These analyses were based on the screened-sample DEG sets.

GO biological process enrichment analysis identified enriched terms in the screened-sample DEG sets from each intestinal segment (Figure 4). Jejunal differentially expressed genes were enriched mainly in nutrient metabolism and sodium ion transport, ileal differentially expressed genes in DNA replication and mitosis-related processes, and cecal differentially expressed genes in extracellular matrix organization, cell adhesion, and tissue remodeling. Only terms with FDR < 0.05 are shown in Figure 4. Complete GO and KEGG enrichment results are provided in Tables S4 and S5, respectively.

### 3.4. Gene Set Enrichment Patterns

**Figure 5:**
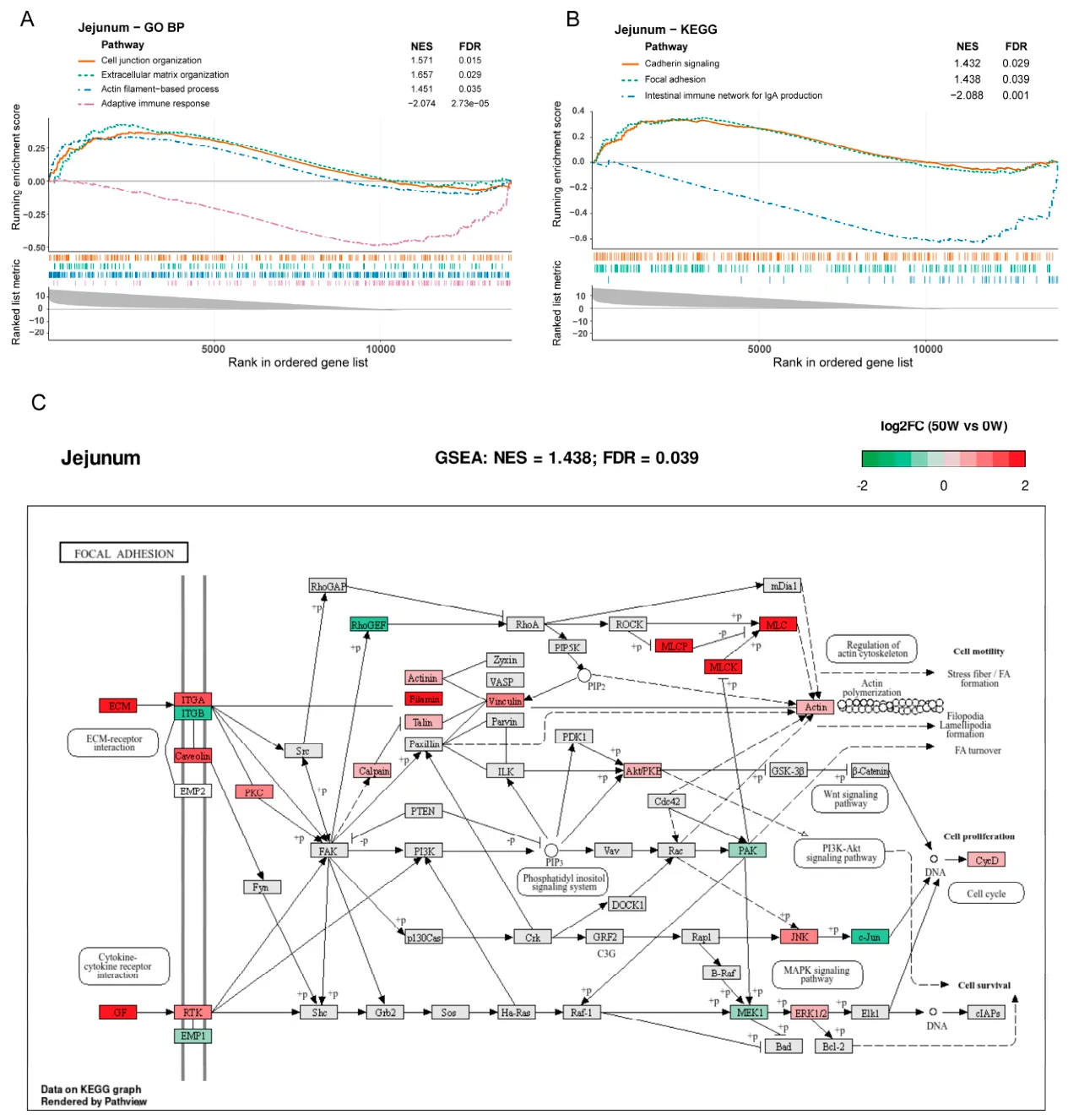
Gene set enrichment analysis (GSEA) of jejunal transcriptomic responses to replacing 50% of dietary soybean meal with walnut cake in Large Diqing Tibetan pigs. (**A**) Enrichment plots for representative Gene Ontology biological process gene sets. (**B**) Enrichment plots for representative Kyoto Encyclopedia of Genes and Genomes (KEGG) pathways. (**C**) KEGG focal adhesion pathway map. Red and green indicate positive and negative log_2_ fold changes, respectively, for the 50W versus 0W contrast. Positive and negative normalized enrichment scores indicate enrichment toward the 50W- and 0W-associated ends of the ranked gene list, respectively. NES, normalized enrichment score; FDR, false discovery rate; 0W, basal-diet control; 50W, group in which walnut cake replaced 50% of dietary soybean meal. The analyses were based on eight retained jejunal samples (0W, *n* = 4; 50W, *n* = 4).

GSEA further revealed distinct enrichment patterns among the three intestinal segments. In the jejunum, GO biological process analysis showed that cell junction organization, extracellular matrix organization, and actin filament-based process were enriched toward the 50W-associated end of the ranked gene list, whereas adaptive immune response was enriched toward the 0W-associated end (Figure 5A). KEGG analysis showed that the cadherin signaling pathway and focal adhesion were enriched toward the 50W-associated end, whereas the intestinal immune network for IgA production was enriched toward the 0W-associated end (Figure 5B). Among these pathways, focal adhesion was significantly enriched toward the 50W-associated end (NES = 1.438, FDR = 0.0391), and the expression changes in genes involved in this pathway are displayed in Figure 5C.

**Figure 6:**
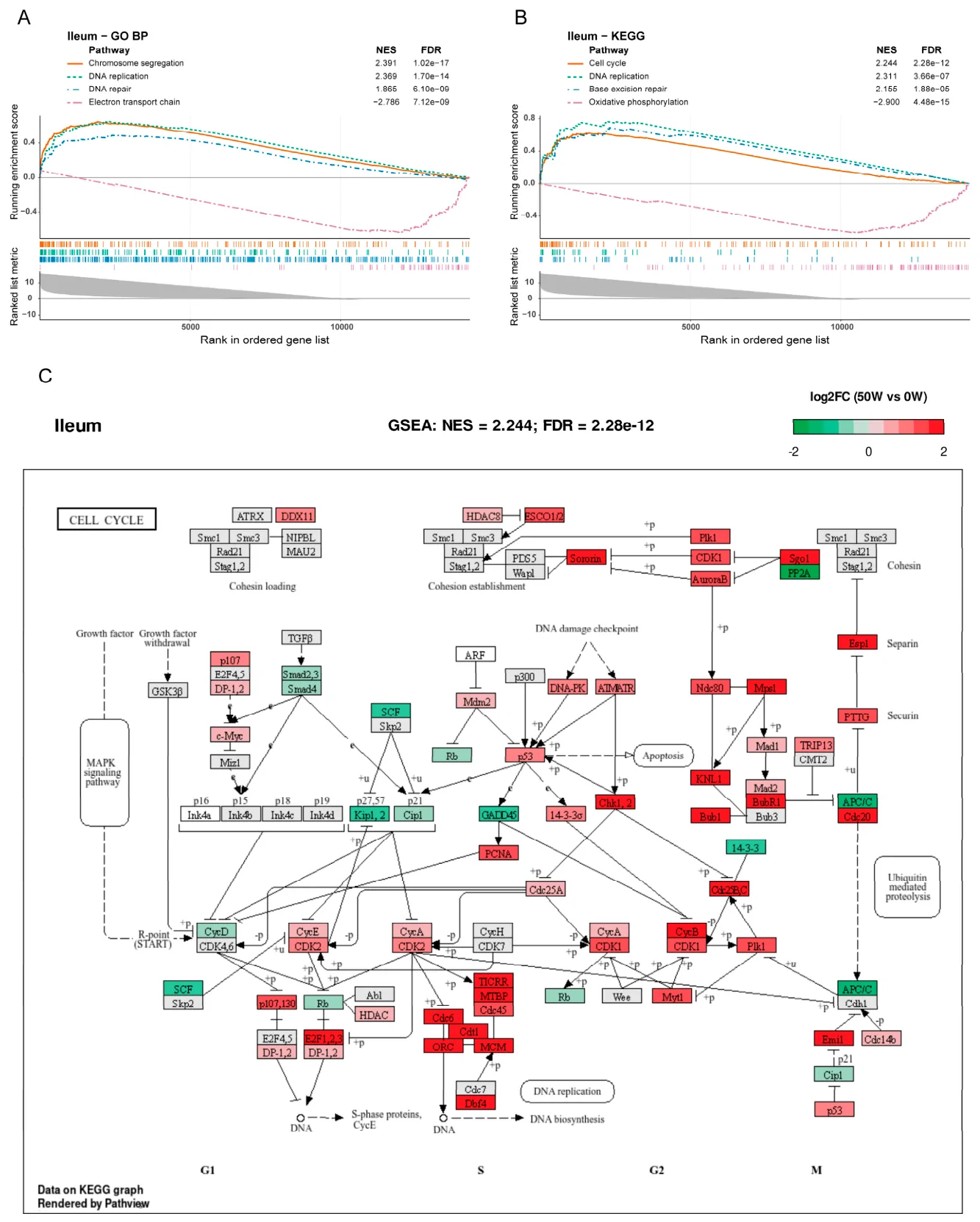
Gene set enrichment analysis (GSEA) of ileal transcriptomic responses to replacing 50% of dietary soybean meal with walnut cake in Large Diqing Tibetan pigs. (**A**) Enrichment plots for representative Gene Ontology biological process gene sets. (**B**) Enrichment plots for representative Kyoto Encyclopedia of Genes and Genomes (KEGG) pathways. (**C**) KEGG cell cycle pathway map. Red and green indicate positive and negative log_2_ fold changes, respectively, for the 50W versus 0W contrast. Positive and negative normalized enrichment scores indicate enrichment toward the 50W- and 0W-associated ends of the ranked gene list, respectively. NES, normalized enrichment score; FDR, false discovery rate; 0W, basal-diet control; 50W, group in which walnut cake replaced 50% of dietary soybean meal. These analyses were based on all 12 ileal samples (0W, *n* = 6; 50W, *n* = 6).

In the ileum, chromosome segregation, DNA replication, and DNA repair were enriched toward the 50W-associated end, whereas the electron transport chain was enriched toward the 0W-associated end in the GO biological process analysis (Figure 6A). Consistently, KEGG analysis showed that the cell cycle, DNA replication, and base excision repair pathways were enriched toward the 50W-associated end, whereas oxidative phosphorylation was enriched toward the 0W-associated end (Figure 6B). Among these pathways, the cell cycle pathway showed strong enrichment toward the 50W-associated end (NES = 2.244, FDR = 2.28 × 10^−12^), and the expression changes in genes involved in this pathway are displayed in Figure 6C.

**Figure 7:**
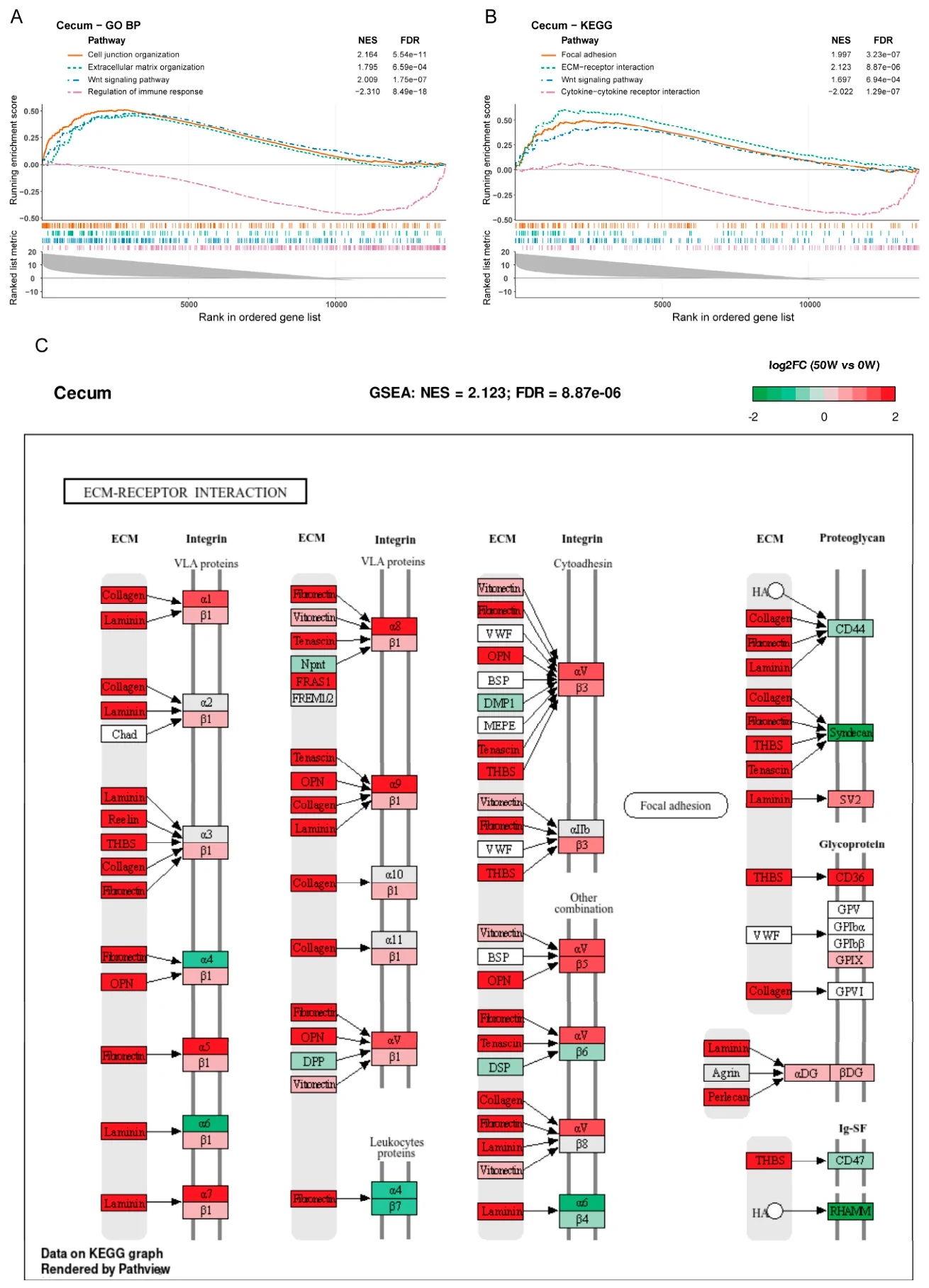
Gene set enrichment analysis (GSEA) of cecal transcriptomic responses to replacing 50% of dietary soybean meal with walnut cake in Large Diqing Tibetan pigs. (**A**) Enrichment plots for representative Gene Ontology biological process gene sets. (**B**) Enrichment plots for representative Kyoto Encyclopedia of Genes and Genomes (KEGG) pathways. (**C**) KEGG extracellular matrix (ECM)–receptor interaction pathway map. Red and green indicate positive and negative log_2_ fold changes, respectively, for the 50W versus 0W contrast. Positive and negative normalized enrichment scores indicate enrichment toward the 50W- and 0W-associated ends of the ranked gene list, respectively. NES, normalized enrichment score; FDR, false discovery rate; ECM, extracellular matrix; 0W, basal-diet control; 50W, group in which walnut cake replaced 50% of dietary soybean meal. The analyses were based on 10 retained cecal samples (0W, *n* = 6; 50W, *n* = 4).

In the cecum, cell junction organization, extracellular matrix organization, and the Wnt signaling pathway were enriched toward the 50W-associated end, whereas regulation of immune response was enriched toward the 0W-associated end in the GO biological process analysis (Figure 7A). KEGG analysis showed that focal adhesion, extracellular matrix (ECM)–receptor interaction, and the Wnt signaling pathway were enriched toward the 50W-associated end, whereas cytokine–cytokine receptor interaction was enriched toward the 0W-associated end (Figure 7B). Among these pathways, ECM–receptor interaction showed strong enrichment toward the 50W-associated end (NES = 2.123, FDR = 8.87 × 10^−6^), and the expression changes of genes involved in this pathway are displayed in Figure 7C. Complete screened-sample GSEA results for all GO categories and KEGG pathways are provided in Tables S6 and S7, respectively.

Full-sample GSEA showed moderate concordance with the screened-sample analysis in the jejunum (Pearson r = 0.605 for GO biological process and 0.591 for KEGG) and strong concordance in the cecum (r = 0.904 and 0.912, respectively). All three representative jejunal KEGG pathways and the representative cecal GO and KEGG pathways retained their enrichment directions and significance in the full-sample analyses, whereas the three 50W-associated jejunal GO terms no longer met the FDR threshold. Across the six pen omissions for each intestinal segment, 98.1%, 98.2%, and 98.7% of the gene sets significant in the full-sample jejunal, ileal, and cecal analyses, respectively, retained the same NES direction after every omission. Complete results are provided in Tables S6–S9.

### 3.5. RT-qPCR Assessment of Selected Differentially Expressed Genes

**Figure 8:**
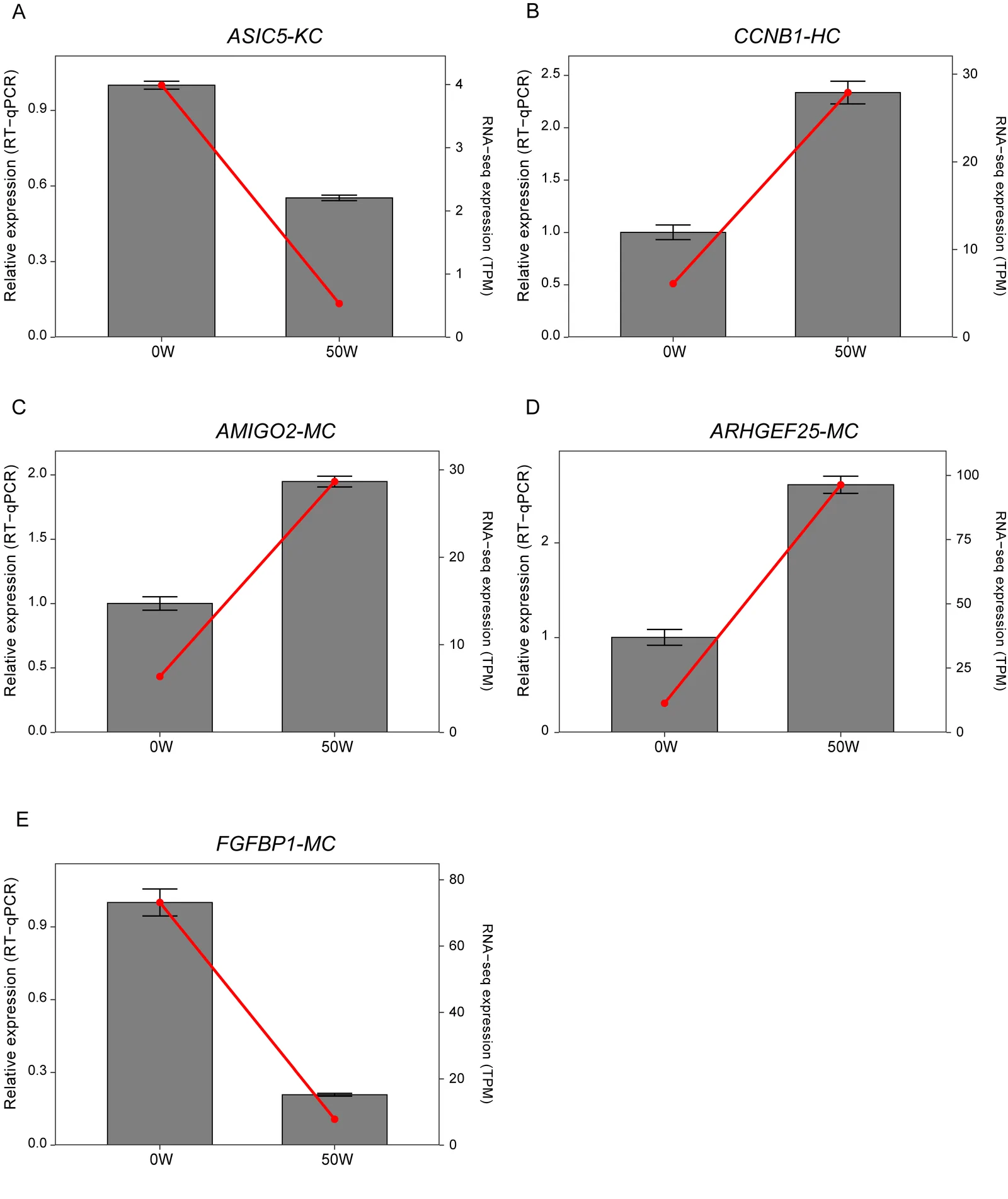
Comparison of RT-qPCR and RNA-seq expression trends for five selected genes in the intestine of Large Diqing Tibetan pigs. (**A**) *ASIC5*-KC; (**B**) *CCNB1*-HC; (**C**) *AMIGO2*-MC; (**D**) *ARHGEF25*-MC; and (**E**) *FGFBP1*-MC. Gray bars show relative expression determined by RT-qPCR using the 2^−ΔΔCt^ method, presented as mean ± standard deviation (*n* = 3 pigs per treatment, with one pig selected from each pen); red lines show the mean RNA-seq expression levels in transcripts per million (TPM) calculated from the same pigs. The left and right y-axes represent RT-qPCR and RNA-seq expression levels, respectively. KC, jejunum; HC, ileum; MC, cecum. 0W, basal-diet control; 50W, group in which walnut cake replaced 50% of dietary soybean meal.

For all five selected genes, RT-qPCR and the corresponding RNA-seq TPM group means showed concordant directions of expression change (Figure 8).

## 4. Discussion

Replacing 50% of dietary soybean meal with walnut cake was associated with higher villus-related indices in the jejunum and ileum and greater crypt depth and mucosal thickness in the cecum. Functional enrichment analysis of DEGs and GSEA further showed that the associated biological processes differed among segments. Full-sample sensitivity analyses showed that the threshold-defined DEG results in the jejunum and cecum were sensitive to sample inclusion, whereas GSEA concordance was strong in the cecum and moderate in the jejunum. Together, these findings describe the morphological and transcriptomic responses to the dietary treatment in the jejunum, ileum, and cecum.

The jejunum is a major site of nutrient digestion and absorption, and villus height, villus width, and the villus height-to-crypt depth ratio may reflect the absorptive interface and structural integrity of the intestinal mucosa [30]. All three indices were higher in the 50W group, whereas crypt depth did not differ, indicating a primarily villus-related response that may reflect expansion of the absorptive interface. Jejunal DEGs were mainly enriched in nutrient metabolism and sodium ion transport, suggesting changes in local nutrient handling and ion transport. GSEA further showed 50W-associated enrichment of cell junction organization, extracellular matrix organization, actin filament-based process, cadherin signaling, and focal adhesion. These patterns pointed to alterations in cell junctions, cell–extracellular matrix interactions, and cytoskeletal organization. Previous research has shown that intestinal epithelial cytoskeletal dynamics contribute to cell mechanics and barrier integrity [33]. Taken together, the jejunal response may involve coordinated changes in the villus absorptive interface and epithelial organization.

Ileal morphological changes were similar to those in the jejunum. Villus height and width were higher in the 50W group, whereas crypt depth and the villus height-to-crypt depth ratio did not differ. Ileal DEGs were mainly enriched in DNA replication and mitotic processes, which are closely associated with intestinal epithelial cell proliferation and renewal [34]. GSEA further showed 50W-associated enrichment of chromosome segregation, DNA replication, DNA repair, cell cycle, and base excision repair. These findings were consistent with the DEG-based enrichment results, indicating that DNA replication and cell-cycle-related processes were an important feature of the ileal transcriptomic response. Dietary effects on the ileal epithelium do not necessarily occur in parallel across molecular, cellular, and morphological levels. In finishing pigs of comparable initial body weight, dietary fiber source altered ileal epithelial cell composition and nutrient-sensing and transport-related gene expression without affecting villus or crypt length or the Ki-67 proliferation index [35]. The concurrent cell-cycle-related enrichment and greater villus dimensions observed here therefore suggest a coordinated local response to the replacement diet, but do not necessarily indicate enhanced nutrient absorption. Similarly, finishing pigs divergent in residual feed intake showed only limited differences in intestinal morphology and function, suggesting that local intestinal traits contribute little to variation in feed efficiency [36]. This agrees with the absence of differences in final body weight and average daily gain between the 0W and 50W groups in the same feeding trial [13]. Thus, the ileal changes likely reflect local adaptation to the replacement diet rather than improved overall growth performance. Their long-term physiological significance may depend on the coordination of epithelial renewal, differentiation, and maturation.

The cecum lacks the typical villous architecture of the small intestine and has distinct hindgut characteristics [37]. Cecal mucosal thickness and crypt depth were higher in the 50W group, whereas muscularis mucosae and muscularis propria thicknesses did not differ. Thus, the morphological response was concentrated in the mucosa and crypts. Cecal DEGs were enriched mainly in extracellular matrix organization, cell adhesion, and tissue remodeling, while the screened-sample GSEA further showed enrichment of cell junction organization, focal adhesion, ECM–receptor interaction, and Wnt signaling. Although the number of threshold-defined cecal DEGs was sensitive to sample inclusion, the full-sample GSEA was strongly concordant with the screened-sample analysis, with the principal ECM-, adhesion-, Wnt-, and immune-related enrichment patterns retained in the same directions. Wnt/β-catenin signaling is involved in intestinal stem cell self-renewal and epithelial regeneration [38], and the concordance between the morphological and transcriptomic changes further points to remodeling of the crypt–mucosal compartment. In finishing pigs, dietary fiber has been shown to alter cecal microbial fiber degradation and short-chain fatty acid profiles together with the expression of cecal genes involved in short-chain fatty acid sensing and transport [39]. In growing pigs, fibrous corn coproducts were also associated with changes in the colonic mucosa-associated microbiota and host genes related to cell structure and extracellular-matrix remodeling [40]. In this context, the increased cecal mucosal thickness and crypt depth, together with the retained ECM-, adhesion-, and Wnt-related GSEA patterns, may reflect a coordinated structural and molecular adaptation of the cecum to the replacement diet within its distinct fermentative environment.

Further comparison of the functional enrichment results showed that the jejunum and cecum shared enrichment of terms related to cell junctions, extracellular matrix organization, and adhesion. However, the jejunum also showed enrichment related to nutrient metabolism, ion transport, actin cytoskeletal organization, and cadherin signaling, whereas the cecum showed prominent enrichment related to ECM–receptor interaction and Wnt signaling. By contrast, the ileal enrichment pattern was markedly different and mainly involved DNA replication, chromosome segregation, DNA repair, and cell-cycle-related processes. GSEA also showed enrichment toward the 0W-associated end for immune-related gene sets in the jejunum and cecum and energy metabolism-related gene sets in the ileum. Related nutritional studies in pigs have also reported region-dependent intestinal transcriptional responses. Altering feeding frequency produced different metabolic gene expression patterns in the ileal and colonic mucosa [41], while replacement of soybean meal with spray-dried plasma resulted in substantially more DEGs in the jejunum than in the ileum and distinct pathway enrichment between the two segments [42]. Although these studies involved different interventions and sampled intestinal sites, their findings are consistent with the present observation that the predominant response patterns differed among intestinal segments. To the best of our knowledge, directly comparable evidence regarding walnut cake-associated transcriptomic responses across the porcine jejunum, ileum, and cecum remains limited.

Overall, replacing 50% of dietary soybean meal with walnut cake was associated with intestinal morphological and transcriptomic changes in Large Diqing Tibetan pigs: the jejunum was characterized mainly by changes in villus-related morphological indices, nutrient metabolism, and ion transport; the ileum by changes in villus architecture, DNA replication, and processes related to epithelial renewal; and the cecum by changes in crypt and mucosal morphology, extracellular matrix organization, and cell adhesion. These findings provide a basis for further evaluating this dietary replacement strategy in Large Diqing Tibetan pigs.

Because corn, soybean oil, and supplemental L-lysine were adjusted to maintain broadly comparable dietary energy, crude protein, and total lysine levels, the present comparison is between two complete dietary formulations. The 50W diet also had a higher crude fiber content than the 0W diet (Table 1). Accordingly, the observed intestinal morphological and transcriptomic responses should be interpreted in the context of the overall dietary replacement strategy rather than attributed to walnut cake or any single dietary component. In addition, each treatment included only three independent pens, and sample screening further reduced pen representation in the jejunal and cecal analyses. Although the sensitivity analyses showed broad consistency in the principal pathway-enrichment patterns, they cannot compensate for the limited number of independent pens in the original design. Further studies with more independent pens and direct functional measurements are needed to confirm these findings.

## 5. Conclusions

Replacing 50% of dietary soybean meal with walnut cake was associated with intestinal morphological and transcriptomic responses in Large Diqing Tibetan pigs. The jejunum and ileum showed higher villus-related indices, with transcriptomic responses related mainly to nutrient metabolism, ion transport, and epithelial organization in the jejunum and to DNA replication, the cell cycle, and epithelial renewal in the ileum. The cecum showed greater crypt depth and mucosal thickness, together with changes related to extracellular matrix organization and cell adhesion. The principal pathway-level patterns were broadly supported by the full-sample and leave-one-pen-out sensitivity analyses, providing a basis for further evaluation of this dietary replacement strategy.

## Figures and Tables

**Table 1:** Dietary composition and nutrient levels (air-dry basis).

Item	Control Group (0W)	Walnut Cake Replacement Group (50W)
Ingredient composition (%)		
Corn	67.60	62.42
Soybean meal	12.00	6.00
Wheat bran	14.90	14.90
Soybean oil	3.00	0.30
Walnut cake	0.00	13.74
L-Lysine (78.8%)	0.00	0.14
Premix ^1^	2.50	2.50
Total	100.00	100.00
Nutrient levels ^2^		
Crude protein (%)	13.52	13.53
Crude fiber (%)	2.76	6.36
Ether extract (%)	6.18	4.36
Digestible energy (MJ/kg)	13.85	13.81
Calcium (%)	0.74	0.79
Available phosphorus (%)	0.34	0.33
Lysine (%)	0.94	0.94
Tryptophan (%)	0.15	0.14
Methionine + cystine (%)	0.50	0.55
Threonine (%)	0.48	0.43
Isoleucine (%)	0.48	0.45
Sodium chloride (%)	0.20	0.20

^1^ The premix provided the following per kilogram of complete diet: vitamin A, 6500 IU; vitamin D_3_, 2000 IU; vitamin E, 42 IU; vitamin K_3_, 2.0 mg; vitamin B_1_, 2.0 mg; vitamin B_2_, 6.4 mg; vitamin B_6_, 3.0 mg; vitamin B_12_, 25.0 μg; nicotinic acid, 1.2 mg; pantothenic acid, 20.0 mg; folic acid, 1.2 mg; biotin, 160.0 μg; nicotinamide, 25 mg; Fe (as ferrous sulfate), 100 mg; Cu (as copper sulfate), 25 mg; Mn (as manganese sulfate), 50 mg; Zn (as zinc sulfate), 80 mg; I (as potassium iodide), 0.14 mg; Se (as sodium selenite), 0.15 mg; methionine, 123.1 mg; L-lysine, 3607.5 mg; and choline chloride, 250 mg. ^2^ Nutrient levels are expressed on an air-dry basis. Digestible energy and the contents of crude fiber, ether extract, available phosphorus, lysine, tryptophan, methionine plus cystine, threonine, and isoleucine are calculated values, whereas the crude protein and calcium contents are analyzed values.

**Table 2:** Primer sequences used for reverse transcription quantitative PCR (RT-qPCR).

Gene	Primer Sequence (5′→3′)	Product Length (bp)
*GAPDH*	F: GACATCAAGAAGGTGGTGAAGCA	177
	R: GTCGTACCAGGAAATGAGCTTGA	
*AMIGO2*	F: TGAACAGATCCCATGCCCAC	108
	R: GGCACGGTGTCAGATAGAGG	
*ARHGEF25*	F: AAGATAACACTGCGAAGCC	225
	R: TGAAGCCGAGATACTCCC	
*FGFBP1*	F: TCATCACCCAAGACCAAGCC	235
	R: ACCCTGGTCTTCAGGACACT	
*ASIC5*	F: CCCAAGGACTTCTGCTCACT	221
	R: AACACCTTGCGAGTTCTGCT	
*CCNB1*	F: TCTGCTGCAACCTCTAAGCC	199
	R: TCTCTGGCTGGGGCTCTAAT	

## Data Availability

The RNA-seq data presented in this study have been deposited in the Genome Sequence Archive (GSA) under accession number CRA044706 and are scheduled for public release on 30 September 2026. Other data presented in this study are available from the corresponding author upon reasonable request.

## Supplementary Materials

The following supporting information can be downloaded at: https://www.mdpi.com/article/10.3390/biology15181636/s1, Figure S1: Unsupervised hierarchical clustering of jejunal, ileal, and cecal RNA-seq samples before sample screening; Figure S2: Principal component analysis of RNA-seq samples before sample screening; Table S1: Nutrient composition of walnut cake (air-dry basis); Table S2: Summary of RNA-seq quality control and genome alignment statistics; Table S3: RNA-seq samples excluded after hierarchical-clustering-based sample screening and distribution of retained samples across pens; Table S4: Complete Gene Ontology enrichment results for differentially expressed genes from the screened-sample analyses in the jejunum, ileum, and cecum; Table S5: Complete Kyoto Encyclopedia of Genes and Genomes pathway enrichment results for differentially expressed genes from the screened-sample analyses in the jejunum, ileum, and cecum; Table S6: Complete GO-based GSEA results from the screened-sample analyses of all three intestinal segments and the full-sample sensitivity analyses of the jejunum and cecum; Table S7: Complete KEGG-based GSEA results from the screened-sample analyses of all three intestinal segments and the full-sample sensitivity analyses of the jejunum and cecum; Table S8: Full-sample differential-expression results and screened-versus-full comparison metrics for the jejunum and cecum; Table S9: Leave-one-pen-out sensitivity analysis of pathway-level transcriptomic results in the jejunum, ileum, and cecum; Table S10: Mean differences and 95% confidence intervals for intestinal morphometric outcomes between the 50W and 0W groups based on pen-level observations.
