# Supplementary material for: Morphological and Transcriptomic Responses of Three Intestinal Segments Following the Replacement of 50% of Dietary Soybean Meal with Walnut Cake in Finishing Large Diqing Tibetan Pigs
Source: Biology (Basel). 2026 Sep 16;15(18):1636. doi: 10.3390/biology15181636 (PMC13604574; doi:10.3390/biology15181636)
Supplement: Supplementary file 1 [file biology-15-01636-s001.zip › Figure S1. Unsupervised hierarchical clustering of jejunal, ileal, and cecal RNA-seq samples before sample screening.pdf]

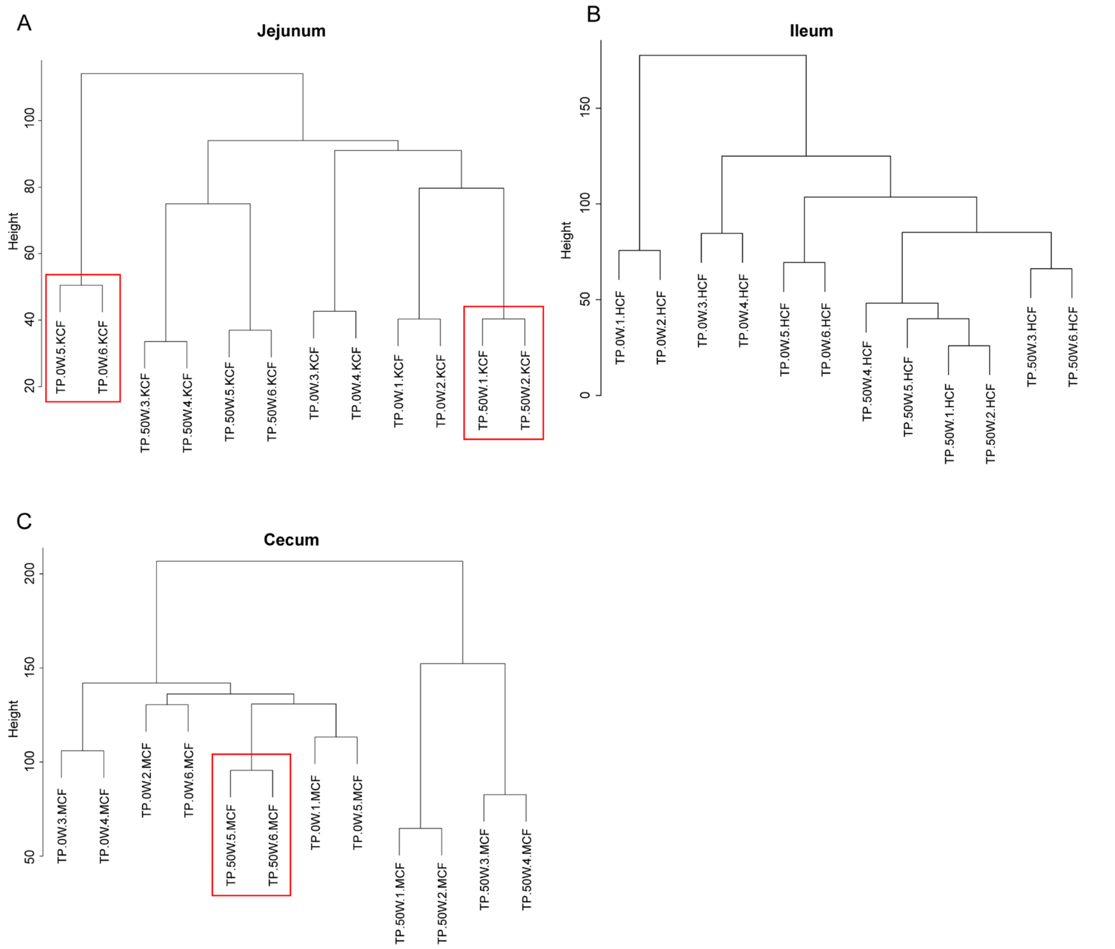

Figure S1. Unsupervised hierarchical clustering of jejunal, ileal, and cecal RNA-seq samples before sample screening. (A–C) Hierarchical clustering dendrograms of the jejunal, ileal, and cecal samples, respectively. Red boxes indicate samples that were not retained in the screened-sample analysis.
