# Supplementary material for: Morphological and Transcriptomic Responses of Three Intestinal Segments Following the Replacement of 50% of Dietary Soybean Meal with Walnut Cake in Finishing Large Diqing Tibetan Pigs
Source: Biology (Basel). 2026 Sep 16;15(18):1636. doi: 10.3390/biology15181636 (PMC13604574; doi:10.3390/biology15181636)
Supplement: Supplementary file 1 [file biology-15-01636-s001.zip › Table S2. Summary of RNA-seq quality control and genome alignment statistics.pdf]

Table S2. Summary of RNA-seq quality control and genome alignment statistics.

Tissue	Group	Sample	Q20 (%)	Q30 (%)	GC (%)	Mapping rate (%)	Clean reads (M)	Unique mapping (%)	Duplication (%)
Jejunum	0W	0W-1	99.31	96.17	51.24	96.58	37.66	92.55	13.09
		0W-2	99.24	95.80	51.24	96.37	38.88	92.28	12.85
		0W-3	99.35	96.34	50.71	95.50	36.80	91.75	17.66
		0W-4	99.21	95.63	51.25	96.12	32.64	92.61	14.17
		0W-5	99.22	95.71	50.69	96.69	42.20	93.85	11.66
		0W-6	99.22	95.66	50.63	96.55	44.04	93.60	12.36
	50W	50W-1	99.21	95.61	50.80	95.80	36.18	92.56	14.08
		50W-2	99.34	96.16	50.61	96.08	38.08	92.99	17.07
		50W-3	99.18	95.44	51.03	96.57	32.91	93.73	12.56
		50W-4	99.20	95.56	51.01	96.50	33.84	93.51	12.70
		50W-5	99.22	95.69	51.33	96.39	38.39	93.17	12.93
		50W-6	99.21	95.65	51.20	96.72	37.87	93.75	11.89
Ileum	0W	0W-1	99.45	98.25	45.36	97.39	49.99	95.02	20.08
		0W-2	99.31	97.78	50.86	98.13	51.51	95.20	13.50
		0W-3	99.32	97.79	48.81	97.72	46.63	94.80	13.43
		0W-4	99.37	97.96	46.30	97.64	50.00	93.49	22.75
		0W-5	99.28	97.67	48.66	97.38	47.87	94.01	24.75
		0W-6	99.26	97.61	48.36	97.51	51.34	94.13	31.22
	50W	50W-1	99.18	97.35	50.17	97.37	51.99	94.67	13.85
		50W-2	99.20	97.42	50.77	97.21	48.39	94.53	11.52
		50W-3	99.24	97.54	50.85	97.54	49.50	94.70	12.19
		50W-4	99.29	97.70	48.71	97.67	49.06	95.09	12.37

Tissue	Group	Sample	Q20 (%)	Q30 (%)	GC (%)	Mapping rate (%)	Clean reads (M)	Unique mapping (%)	Duplication (%)
		50W-5	99.27	97.64	47.84	96.56	41.46	94.06	11.57
		50W-6	99.15	97.22	50.27	97.50	48.04	94.68	13.53
Cecum	0W	0W-1	99.41	98.11	45.86	97.76	44.80	92.97	29.23
		0W-2	99.20	97.42	51.43	97.22	48.71	90.47	16.18
		0W-3	99.20	97.44	53.78	97.13	46.86	91.87	12.58
		0W-4	99.24	97.54	49.66	97.23	46.96	92.91	20.29
		0W-5	99.37	97.97	45.29	97.49	48.65	92.84	28.97
		0W-6	99.42	98.12	48.33	95.30	49.21	91.70	20.04
	50W	50W-1	99.28	97.69	48.88	97.36	49.31	93.45	30.20
		50W-2	99.21	97.47	48.75	97.45	48.58	93.79	25.09
		50W-3	99.43	98.16	45.84	89.98	47.41	85.19	37.02
		50W-4	99.33	97.81	46.91	97.29	51.86	92.07	36.14
		50W-5	99.20	97.54	57.10	95.64	51.03	52.33	43.67
		50W-6	99.34	97.82	45.50	97.38	48.62	92.52	37.83

Note: 0W indicates the control group, and 50W indicates the walnut cake replacement group in which 50% of dietary soybean meal was replaced with walnut cake. Q20, Q30, GC, clean reads, and duplication rates were obtained from the fastp quality-control results. Clean reads indicate the number of reads retained after quality filtering and are expressed in million reads (M). Mapping rate represents the overall alignment rate generated by HISAT2. Unique mapping was calculated from the HISAT2-generated alignment files as the percentage of uniquely mapped primary reads among total primary reads. Duplication represents the sequence-duplication rate estimated by fastp.
