# Supplementary material for: Morphological and Transcriptomic Responses of Three Intestinal Segments Following the Replacement of 50% of Dietary Soybean Meal with Walnut Cake in Finishing Large Diqing Tibetan Pigs
Source: Biology (Basel). 2026 Sep 16;15(18):1636. doi: 10.3390/biology15181636 (PMC13604574; doi:10.3390/biology15181636)
Supplement: Supplementary file 1 [file biology-15-01636-s001.zip › Figure S2. Principal component analysis of RNA-seq samples before sample screening.pdf]

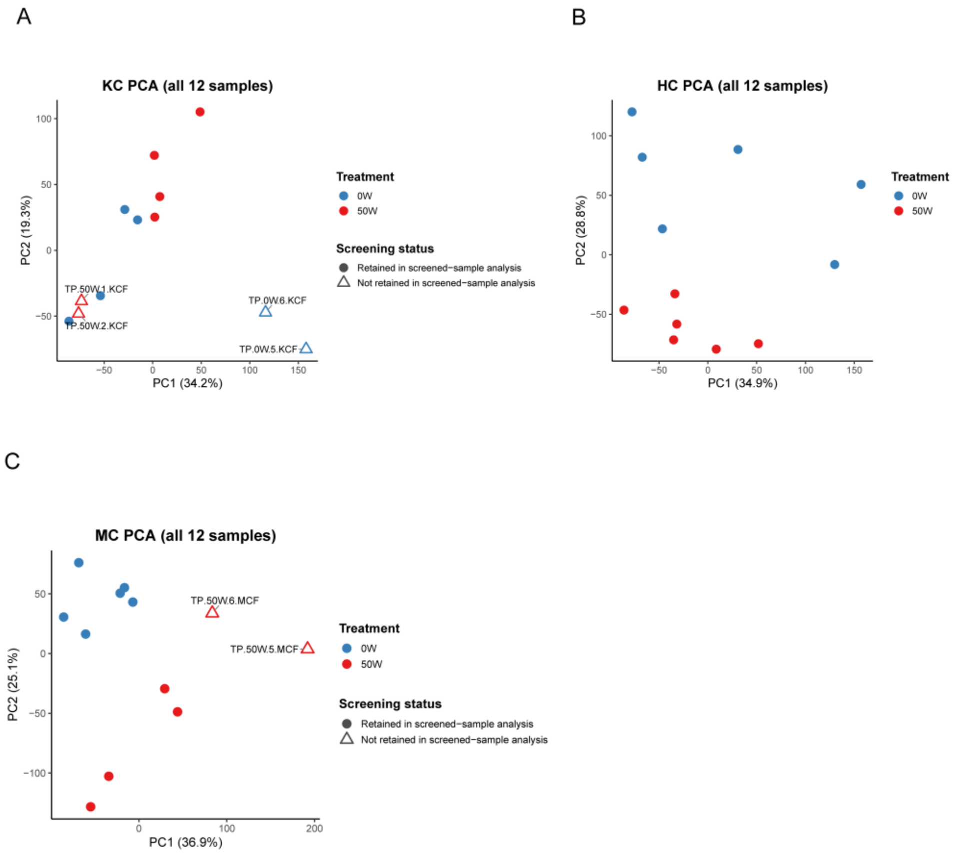

Figure S2. Principal component analysis of RNA-seq samples before sample screening. (A–C) Principal component analysis of all 12 originally sequenced jejunal, ileal, and cecal samples, respectively. Colors indicate dietary treatments. Open triangles and sample labels indicate samples that were not retained in the screened-sample analysis.
