# Supplementary material for: Morphological and Transcriptomic Responses of Three Intestinal Segments Following the Replacement of 50% of Dietary Soybean Meal with Walnut Cake in Finishing Large Diqing Tibetan Pigs
Source: Biology (Basel). 2026 Sep 16;15(18):1636. doi: 10.3390/biology15181636 (PMC13604574; doi:10.3390/biology15181636)
Supplement: Supplementary file 1 [file biology-15-01636-s001.zip › Table S1. Nutrient composition of walnut cake (air-dry basis).pdf]

Table S1. Nutrient composition of walnut cake (air-dry basis).

Item	Content (%)
Crude protein	22.62±0.45
Crude fat	8.17±0.11
Crude ash	2.78±0.05
Crude fiber	29.37±0.54
Neutral detergent fiber	49.26±0.82
Acid detergent fiber	33.14±0.79
Calcium	0.45±0.01
Total phosphorus	0.52±0.18

The nutrient composition of walnut cake was previously reported [13].
