# Supplementary material for: Morphological and Transcriptomic Responses of Three Intestinal Segments Following the Replacement of 50% of Dietary Soybean Meal with Walnut Cake in Finishing Large Diqing Tibetan Pigs
Source: Biology (Basel). 2026 Sep 16;15(18):1636. doi: 10.3390/biology15181636 (PMC13604574; doi:10.3390/biology15181636)
Supplement: Supplementary file 1 [file biology-15-01636-s001.zip › Table S3. RNA-seq samples excluded after hierarchical-clustering-based sample screening and distribution of retained samples across pens.pdf]

Table S3A. RNA-seq samples excluded following sample screening based on unsupervised hierarchical clustering.

Intestinal segment	Sample ID	Dietary group	Pen ID
Jejunum	TP.0W.5.KCF	0W	0W_P3
Jejunum	TP.0W.6.KCF	0W	0W_P3
Jejunum	TP.50W.1.KCF	50W	50W_P1
Jejunum	TP.50W.2.KCF	50W	50W_P1
Cecum	TP.50W.5.MCF	50W	50W_P3
Cecum	TP.50W.6.MCF	50W	50W_P3

Samples showing distinct sample-level clustering patterns in the unsupervised hierarchical clustering of variance-stabilized expression profiles were excluded from the primary screened-sample analyses. The corresponding sample dendrograms are shown in Figure S1.

Table S3B. Distribution of retained RNA-seq samples across pens.

Treatment	Pen	Jejunum, n	Ileum, n	Cecum, n
0W	P1	2/2	2/2	2/2
0W	P2	2/2	2/2	2/2
0W	P3	2/0	2/2	2/2
50W	P1	2/0	2/2	2/2
50W	P2	2/2	2/2	2/2
50W	P3	2/2	2/2	2/0

Values are presented as the number of initially sequenced samples/the number of samples retained in the screened-sample analysis.
